# The Association between Body Mass Index and Physical Fitness of Normal Weight/Overweight/Obese University Students

**DOI:** 10.3390/ijerph17155391

**Published:** 2020-07-27

**Authors:** Ya-Tzu Kung, Chia-Ming Chang, Fang-Ming Hwang, Shyh-Ching Chi

**Affiliations:** 1 Graduate Institute of Physical Education, National Taiwan Sport University, Taoyuan 33301, Taiwan; ivy022011@gmail.com; 2 Department of Physical Education, Health & Recreation, National Chiayi University, Chiayi County 62103, Taiwan; gr5166@yahoo.com.tw; 3 Department of Education, National Chiayi University, Chiayi County 62103, Taiwan; fmh@mail.ncyu.edu.tw; 4 Department of Sports Training Science-Balls, National Taiwan Sport University, Taoyuan 33301, Taiwan

**Keywords:** BMI, physical fitness, overweight, longitudinal data

## Abstract

This study aimed to apply longitudinal data (in four waves) to examine relationships between body mass index (BMI), flexibility, muscular endurance, and explosive power, and employed a random-intercept panel model (RIPM) to divide the variations of different waves into between- and within-person variations. Furthermore, a multi-group model test was conducted to explore whether an interaction effect existed between sex and these relationships. The data were collected from a university in Taiwan between August 2009 and July 2010, and 3863 freshman and junior students were recruited. Results showed that the between- and within-person relationships between BMI and explosive power, and between BMI and muscular endurance, were negative (independent of sex). The negative between- and within-person associations between BMI and muscular endurance were both invariant with respect to sex. The negative between-person associations between BMI and explosive power were not equivalent for both sexes, yet the within-person associations between BMI and explosive power were equivalent for both sexes. The between-person associations between BMI and flexibility were positive for both sexes, but the within-person associations of these two variables were negative for both sexes. The invariance test confirmed that the positive associations of between-person BMI and between-person flexibility were invariant for both sexes, yet the negative associations of within-person BMI and within-person flexibility were not equivalent for both sexes. Between-and within-person correlations among explosive power, muscular endurance, and flexibility were significantly positive. Only the auto-regressions of BMI and muscular endurance were significant and equivalent for both sexes.

## 1. Introduction

The rates of obesity and being overweight among the young have been increasing in developed and developing countries alike [1]. According to a report released by the World Health Organization [2], in 2016, 18% of children and adolescents worldwide (340 million) were obese or overweight. A study of teenage obesity and socioeconomic status in Taiwan revealed that the overall prevalence of combined obesity and being overweight among 1,875,627 adolescents (aged 10–18 years) was 28.1%. Boys were more likely to be overweight/obese than girls (32.1% vs. 23.6%) [3]. The data demonstrated that adolescent obesity and being overweight have become a relatively severe problem, one increasingly discussed by Taiwan’s Ministry of Education and the general public.

Physical fitness is regarded as an essential indicator for health [4,5,6] and is strongly associated with other positive health indicators for young adults, such as a reduced prevalence of cardiovascular disease risk factors, reduced abdominal obesity, improved mental health and bone health, and decreased all-cause mortality [5]. Research has also indicated that physical fitness is more strongly associated with obesity and being overweight than the risk factors of physical activities and metabolic syndrome [7].

Many countries have examined the relationship between weight status and physical fitness. The governments of countries in both East Asia and the West have consistently determined that obesity and being overweight have a close negative relationship with physical fitness. A systematic review conducted by Rauner et al. [8] revealed that all studies during 2000–2013 demonstrated an inverse relationship between obesity and physical fitness. Compared with individuals with an average weight status, those who were overweight or obese had a lower level of physical fitness, including cardiovascular function, speed, and endurance running [9,10] and the worst performance in nearly all test items, excluding muscular strength [11]. Research by Liao et al. [12] demonstrated that in a large sample of young adults in Taiwan, those with a lower level of body explosive strength and cardiorespiratory endurance have a higher odds ratio (OR) of being overweight and obese. Research concerning children and young adults in China conducted by Zhang et al. [13] indicated that those who were overweight or obese, independent of sex, had inferior performance in all physical fitness tests than those with an average weight status.

Studies examining the relationship between weight status and physical fitness among overweight/obese individuals have mostly used cross-sectional data [1,4,5,6,7,8,9,11,12,13]; that is, the relationship between being overweight/obesity and physical fitness was measured at a single point in time. Such data can reflect between-person relationships but cannot reflect within-person relationships between being overweight/obesity and physical fitness. Accordingly, this study applied longitudinal data collected at several points of time to distinguish the between-person variation in the relationship between overweight/obese weight status and physical fitness from the within-person variation, thus determining whether the relationship of between-person overweight/obese weight status and physical fitness is consistent with the results of preceding studies, as well as the nature of the relationship between within-person overweight/obese weight status and physical fitness. As Mroczek et al. [14] stated, within-person variation is also worth investigating because it provides practitioners and researchers with crucial information about changes in personal life. To our knowledge, no relevant research has used this approach; hence, this study attempted to fill the research gap.

A report by Aires et al. [15] indicated that obese boys and girls have significantly inferior performance as their body mass index (BMI) increases. Artero et al. [16] discovered that boys and girls with an overweight/obese weight status have poorer performance in a 20 m shuttle run, flexed arm hang, standing long jump, and 4 × 10 m shuttle run than those with a normal weight status. Mak et al. [17] reported that overweight/obese boys and girls perform worse in push-ups, sit-ups, and endurance running. Those with overweight/obese weight status exhibit significantly inferior performance in seated forward bend and sit-ups than those with an underweight to normal weight status. The report of Casonatto et al. [18] noted an increased OR between children who are overweight/obese and inferior physical performance (boys: cardiorespiratory fitness OR = 3.64, muscular strength/endurance OR = 1.94; girls: cardiorespiratory fitness OR = 5.03, muscular strength/endurance OR = 2.62).

García-Hermoso et al. [19] discovered an inverted J-curve relationship in the BMI and cardiorespiratory fitness of both boys and girls for adolescents aged 13–15 years, although the parabola’s peak for boys is sharper than that for girls. Lopes et al. [20] determined that, for boys aged 14–15 and 16–17 years, the relationship between BMI and performance in standing long jump, push-ups, and a multistage shuttle run was nonlinear; for girls aged 14–15 and 16–17 years, the relationship between BMI and performance in standing long jump was linear; for girls aged 10–11, 14–15, and 16–17 years, the relationship between BMI and performance in push-ups was nonlinear; and for girls aged 14–15 and 16–17 years, the relationship between BMI and performance in the multistage shuttle run was linear and nonlinear, respectively. Kwieciński et al. [21] revealed that relationships were nonlinear between boys’ BMI and performance in all six physical fitness test items and between BMI and girls’ performance in four of the six tests for adolescents aged 13–16 years. Zhang et al. [13] examined the relationship between weight status and physical fitness of Chinese children and young adults and disclosed that, independent of sex, students with obesity performed poorer in all three selected physical fitness test items than those with an average weight status. In particular, fourth- and eighth-grade boys had large and medium-to-large Cohen’s d values (d = 1.02–1.50; d = 0.76–1.49), respectively, whereas fourth- and eighth-grade girls had medium-to-large and medium values (d = 0.78–0.97; d = 0.51–0.61), respectively.

After a close inspection of these studies, we discovered that the data for boys and girls concerning relationships between weight status and physical fitness were processed separately, and whether relationships between weight status and physical fitness are invariant when the results of boys and girls are examined together—that is, whether an interaction effect exists between sex and physical fitness—is unclear. This information could provide additional insight into the mechanism between weight status and physical fitness. A feasible statistical model to address this problem is necessary for studies of the relationship between weight status and physical fitness.

For these two research gaps, this study proposed a random-intercept panel model (RIPM) to collect four waves of longitudinal data from a university in Taiwan and examine the relationships for university students with normal/overweight/obese weight status between BMI and their flexibility, muscular endurance, and explosive power. The RIPM divided the variation of different waves into between- and within-person variation. Between-person variation resolved the single-point-of-time measurement problem of cross-sectional data, whereas within-person variation explored the relationships between within-person BMI and within-person flexibility, muscular endurance, and explosive power. Therefore, it is hypothesized that there are significant between- and within-level associations between BMI and flexibility, between BMI and muscular endurance, between BMI and explosive power, between flexibility and muscular endurance, between flexibility and explosive power, and between muscular endurance and explosive power. The model was also applied to examine the autoregressive effects of within-person variation, which can be used to investigate the carry-over effects of participants’ BMI, flexibility, muscular endurance, and explosive power. It is hypothesized that BMI, flexibility, muscular endurance, and explosive power will be significantly related to subsequent BMI, subsequent flexibility, subsequent muscular endurance, and subsequent explosive power, respectively. Eventually, a multi-group model test was conducted to explore whether an interaction effect existed between sex and these relationships. It is hypothesized that sex moderates the between- and within-level relationships between BMI and flexibility, between BMI and muscular endurance, between BMI and explosive power, between flexibility and muscular endurance, between flexibility and explosive power, and between muscular endurance and explosive power. This statistical test furnishes more informative results for practitioners and researchers who are interested in the potential mechanism between weight status and physical fitness.

## 2. Materials and Methods

### 2.1. Design and Participants

The data for this study were collected from the Taiwan Youth Physical Fitness Survey conducted by a university in Taiwan to cooperate with a request by the Ministry of Education and the Teaching Excellence Project implemented by the university. The physical fitness testing took place between August 2009 and July 2010, and 3863 freshman and junior students aged between 20–24 years were recruited. Participants were tested in at most four waves 3 months apart. All participants were informed of the instructions for the tests and were asked to sign a consent form. Testers in this study who had completed relevant physical fitness courses and been trained by the Department of Physical Education to conduct physical fitness tests were guided by the principal investigator to administer the tests.

### 2.2. Measures

The test items were conducted in accordance with the requirements of the Taiwan Youth Physical Fitness Survey [22]. Their validity was confirmed by Chiang, Wu, and Shih’s study [23]. This study examined items associated with flexibility, muscular endurance, and explosive power.

#### 2.2.1. Body Mass Index

Body mass index (BMI) is a measure derived from the mass (weight) and height of a person and is defined as the body mass divided by the square of the body height, and is universally expressed in units of kg/m^2^, resulting from mass in kilograms and height in meters [24]. The heights and weights of the participants in each wave were measured. Those with a normal, overweight, and obese weight status were selected for this study; accordingly, students’ BMI was calculated by dividing their weight (kg) by the square of their height (m), and 3126 students with a BMI of not less than 18.5, according to the definition proposed by the Commission on Obesity Definition and Management, Taiwan Centers for Disease Control, were enrolled. Demographic information such as sex, age, year of study, and college were obtained, and sex was adopted as the demographic variable of the interaction effect.

#### 2.2.2. Flexibility

The sit and reach test is used to measure of flexibility of the lower back and hamstring muscles. Each student sitting on the floor with legs stretched out straight ahead. Place one hand on top of the other, then reach forward. The test was scored as the most distant point (nearest centimeter) reach measuring from their toes to their finger tips on the ruler. Before the test, shoes were removed and students were instructed to slowly reach forward with their knees fully extended, as far as possible on the scale. Two trials were recorded, and the better of the two was retained for analysis [22].

#### 2.2.3. Muscular Endurance

The 1 min sit up test as a test for muscular endurance. The test was scored as the number of sit-ups performed in 1 min. Each student begins by sitting comfortably on the mat, he should start with his back straight on the mat with knees bent at 90° and hands crossed on the chest. The technician held the student’s ankles firmly for support and maintained the count. The student’s elbows had to touch the knee with the same side (i.e., left elbow to left knee). After each upward movement, the two sides of scapular returned to touch the mat, but the head did not have to touch it [22].

#### 2.2.4. Explosive Power

A standing long jump is used as a functional test to assess leg power. Each student stood behind a line marked on the ground with feet slightly apart and was instructed to jump horizontally forward as far as possible, taking off with both feet. The student takes off and lands using both feet, swinging the arms and bending the knees to provide forward drive. The distance from the starting line to the heel of the closest foot was recorded. Two trials were recorded, and the better of the two was retained for analysis [22].

### 2.3. Analysis

**Figure 1:**
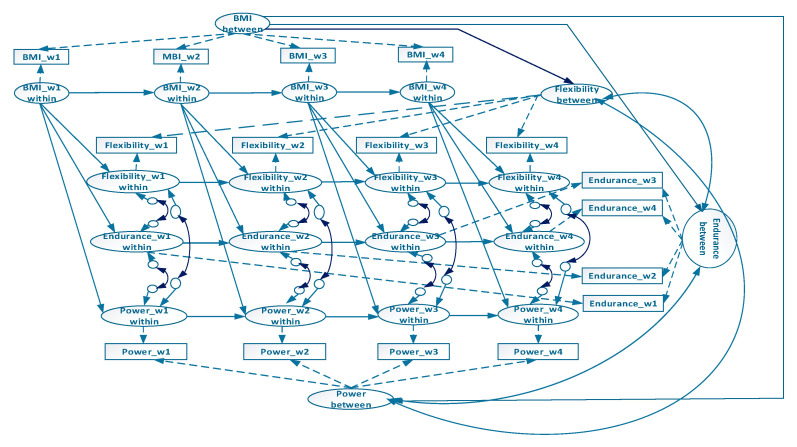
Graphical representation of our four-wave random-intercept panel model on associations between body mass index (BMI), flexibility, muscular endurance, and explosive power (dotted line indicates that the coefficients are fixed to 1.00). w1, wave one; w2, wave two; w3, wave three; w4, wave four.

Structural equation modeling was applied to examine longitudinal relationships concerning participants’ sex, BMI, flexibility, muscular endurance, and explosive power. A RIPM (Figure 1) was developed based on the relationships between BMI, flexibility, muscular endurance, and explosive power. The model distinguished variations in BMI, flexibility, muscular endurance, and explosive power into between- and within-person variations, enabling simultaneous examination of between-persons BMI influence on between-person flexibility, muscular endurance, and explosive power; covariances between between-person flexibility, muscular endurance, and explosive power; within-persons BMI influence on within-person flexibility, muscular endurance, and explosive power; and covariances between within-person flexibility, muscular endurance, and explosive power. Furthermore, autoregressive effects were measured using simple within-person variation to examine the stability of changes in within-person BMI, flexibility, muscular endurance, and explosive power, namely the carry-over effects. Subsequently, a multi-group RIPM for sex was established to inspect the interaction effects of sexes in the relationship of BMI, flexibility, muscular endurance, and explosive power. Data for the overall sample, male sample, female sample, and sex-invariant models were included in the analysis, and the models were processed using Mplus 8.2.

For male participants in the four waves, their analytical data values for BMI, flexibility, muscular endurance, and explosive power were 72.07–90.31%, 75.26–93.32%, 75.20–93.32%, and 75.08–93.20%, respectively, whereas those of the female samples in the four waves were between 74.95% and 89.28%, 82.46% and 99.18%, 82.39% and 99.18%, and 82.39% and 99.18%, respectively (Table 1). The full information maximum likelihood method was applied as an estimation method. Compared with conventional methods (e.g., the listwise deletion method), this method generates estimated values more efficiently and with less bias when data with missing values are used [25].

In addition, the χ^2^ (chi-squared) statistic, root mean square error of approximation (RMSEA), comparative fit index (CFI), and standardized root mean square residual (SRMR) were employed in the goodness-of-fit test. RMSEA values ranging from 0.05 to 0.08 and below 0.05 suggest an acceptable and well-fitting model, respectively [26,27]. CFI values range from 0 to 1; those exceeding 0.90 and 0.95 are considered an acceptable and well-fitting model, respectively. SRMR ranges in value from 0 to 1.00; less than 0.05 indicates a well-fitting model, as high as 0.08 indicates an acceptable model [28,29]. The χ^2^ statistic is considerably sensitive to sample size; however, because this study collected 3126 samples (a relatively large sample size), it only presented the χ^2^ statistic instead of using it as a goodness-of-fit index, as suggested by Jöreskog and Sörbom [30].

The chi-square difference (Δχ^2^) of the constrained and unconstrained models were analyzed to test the multi-group model for the interaction effect of sex; we noted an interaction effect when the difference reached a significant level. SPSS 22.0 was used to obtain descriptive statistics as well as process the *t* test for the sex-independent sample with respect to BMI, flexibility, muscular endurance, and explosive power in each wave. A psych package in the R programming language was used to calculate the intra-class correlation coefficient (ICC(1)). ICC(1) is used to calculate the ratio of between-level variance and total variance [31].

## 3. Results

Table 1 presents the demographics of the participants. Table 2 demonstrates the mean, standard deviation, sample size, and *t* value of the main variables for the male and female participants. In each wave, the male participants had significantly higher BMI, muscular endurance, and explosive power, whereas the female participants had significantly better performance in flexibility.

Table 3 displays the coefficients of BMI, flexibility, muscular endurance, and explosive power with normal/overweight/obese weight status and ICC(1)s. The correlation analysis of the female samples revealed that BMI and flexibility in the four waves were not correlated (−0.03 to 0.06) (Table 3). A zero-to-negative, small correlation existed between BMI and muscular endurance in the four waves (−0.02 to −0.13) (Table 3). BMI and explosive power in the four waves exhibited a negative, small correlation (−0.11 to −0.20) (Table 3), whereas flexibility and muscular endurance and also flexibility and explosive power in the four waves had positive, small correlations (0.09 to 0.19; 0.10 to 0.21) (Table 3). A positive, small-to-moderate correlation was discovered between muscular endurance and explosive power in the four waves (0.24 to 0.39) (Table 3).

BMI in each wave was highly correlated with itself (0.75 to 0.94), and a similar situation existed for flexibility (0.73 to 0.90), muscular endurance (0.62 to 0.79), and explosive power (0.63 to 0.85) (Table 3). The results indicated that BMI, flexibility, muscular endurance, and explosive power of the female samples had substantial stabilities of the rank order of individuals. The ICC(1)s of BMI, flexibility, muscular endurance, and explosive power for the female participants were 0.84, 0.80, 0.70, and 0.72, respectively (Table 3), implying that 84% and 16% demonstrated between-person differences and within-person fluctuations in BMI variation, respectively; 80% and 20% exhibited between-person and within-person flexibility variation, respectively; 70% and 30% had muscular endurance variation with between-person differences and within-person fluctuations, respectively; and 72% and 28% revealed between-person difference and within-person fluctuations in variation in explosive power.

A zero-to-positive, small correlation existed between BMI and flexibility of the male participants in the four waves (−0.02 to 0.07) (Table 3). BMI and muscular endurance of the male participants in the four waves exhibited a negative, small correlation (−0.12 to −0.17), and BMI and explosive power in the four waves had a negative, moderate correlation (−0.24 to −0.36). Male flexibility and muscular endurance and also flexibility and explosive power in the waves showed a positive, small correlation (0.11 to 0.19; 0.13 to 0.17), whereas muscular endurance and explosive power had a positive, small-to-moderate correlation (0.21 to 0.45) (Table 3).

BMI of the male samples in each wave was highly correlated with itself (0.84 to 0.94), and a similar situation existed for flexibility (0.74 to 0.87), muscular endurance (0.57 to 0.70), and explosive power (0.57 to 0.74) (Table 3). The results demonstrated that BMI, flexibility, muscular endurance, and explosive power of the male samples exhibited substantial stabilities of the rank order of individuals. The ICC(1)s of BMI, flexibility, muscular endurance, and explosive power were 0.90, 0.81, 0.65, and 0.67, respectively, suggesting that 90% and 10% had between-person differences and within-person fluctuations, respectively, in BMI variation; 81% and 19% exhibited between-person differences and within-person fluctuations in flexibility; 65% and 35% had between-person differences and within-person fluctuations in muscular endurance; and 67% and 33% revealed between-person difference and within-person fluctuations in explosive power (Table 3).

**Figure 2:**
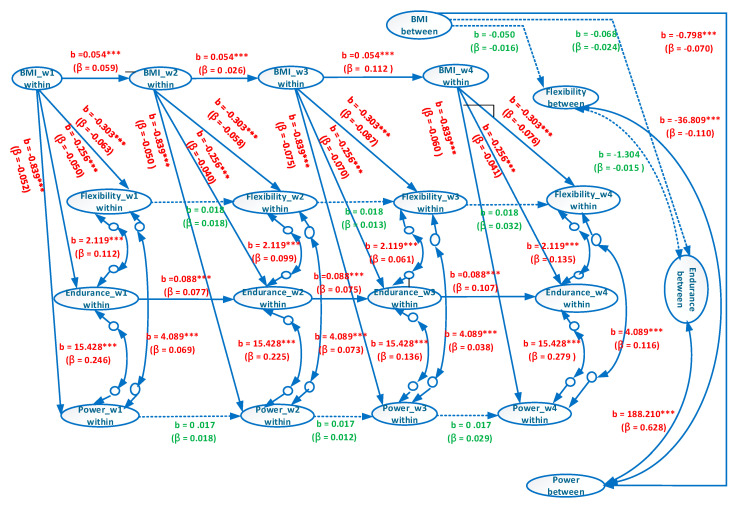
Simplified representation of the estimated random intercept panel model of all samples.

The overall model displays an acceptable goodness of fit (χ^2^(112) = 1575.11, *p* < 0.001, RMSEA = 0.065, CI of RMSEA = (0.062, 0.067), CFI = 0.968, SRMR = 0.078). The estimated coefficients are presented in Figure 2. The male and female models also exhibit an acceptable goodness of fit (χ^2^(112) = 877.66, *p* < 0.001, RMSEA = 0.064, CI of RMSEA = (0.060, 0.068), CFI = 0.962, SRMR = 0.071; χ^2^(112) = 956.26, *p* < 0.001, RMSEA = 0.072, CI of RMSEA = (0.068, 0.076), CFI = 0.956, SRMR = 0.100).

**Figure 3:**
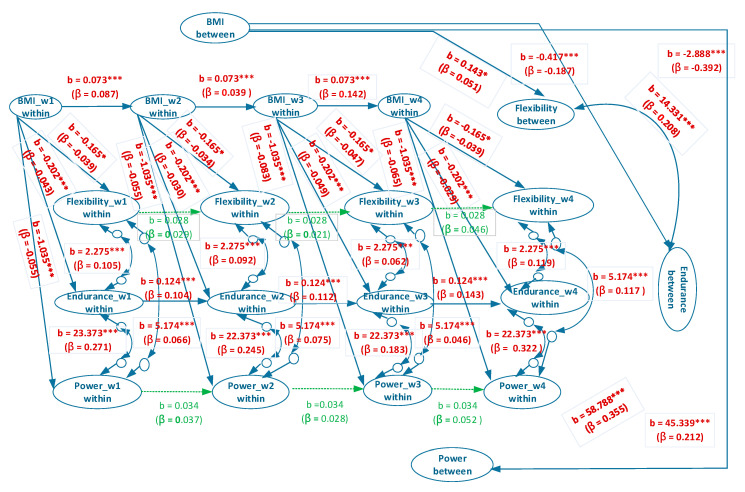
Simplified representation of the estimated random-intercept panel model for male participants.

**Figure 4:**
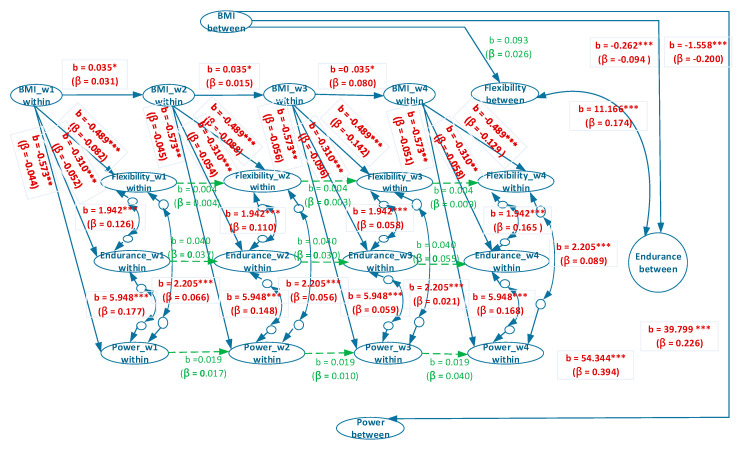
Simplied representation of the estimated random-intercept panel model for female participants.

Figure 3 and Figure 4 provide the estimated coefficients of the overall model and estimated coefficients of the separate male and female models, respectively. In the between-person association between BMI and explosive power, the coefficients of the overall, male, and female models were b = −0.798, β = −0.070, *p* < 0.001; b = −2.888, β = −0.392, *p* < 0.001; and b = −1.558, β = −0.200, *p* < 0.001, respectively (Figure 3 and Figure 4). These results indicated that for university students with a normal/overweight/obese status, a higher BMI suggests a worse performance in explosive power. However, the coefficients of the overall model were evidently lower than those of the individual male and female models, and a Simpson’s paradox occurs.

For the between-person association between BMI and muscular endurance, the coefficients of the overall, male, and female models were b= −0.068, β = −0.024, *p* > 0.05; b = −0.417, *β* = −0.187, *p* < 0.001; and b = −0.262, β = −0.094, *p* < 0.001, respectively (Figure 3 and Figure 4). Thus, a Simpson’s paradox also occurred in this relationship. The result for the overall model did not correspond to those of the male and female models. The overall model suggested that the between-person association between BMI and muscular endurance did not hold. However, the male and female models implied that a higher between-person BMI corresponded to poorer between-person muscular endurance.

In the between-person relationship between BMI and flexibility, the coefficients of the overall, male, and female models were b = −0.050, β = −0.016, *p* > 0.05; b = 0.143, β = 0.051, *p* < 0.05; and b = 0.093, β = 0.026, *p* > 0.05, respectively (Figure 3 and Figure 4). A Simpson’s paradox was also discovered for the overall model. The results of the overall and female models demonstrated that between-person BMI had no correlation with between-person flexibility, whereas that of the male model indicated that higher between-person BMI corresponded to higher between-person flexibility.

In the between-person association between flexibility and muscular endurance, the coefficients of the overall, male, and female models were b = −1.304, β = −0.015, *p* > 0.05; b = 14.331, β = 0.208, *p* < 0.001; and b = 11.166, β = 0.174, *p* < 0.001, respectively (Figure 3 and Figure 4). The between-person correlation between flexibility and muscular endurance was not significant. However, the results of the male and female models revealed a significantly positive between-person correlation between flexibility and muscular endurance. This result indicated that Simpson’s paradox was present in the overall model.

In the between-person association between flexibility and explosive power, the coefficients of the overall, male, and female models were b = −36.809, β = −0.110, *p* < 0.001; b = 45.339, β = 0.212, *p* < 0.001; and b = 39.799, β = 0.226, *p* < 0.001, respectively (Figure 3 and Figure 4). A Simpson’s paradox again occurs in the overall model. The result of the overall model implied that between-person flexibility and explosive power were significantly and negatively correlated; in other words, a higher level of between-person flexibility corresponded to a lower level of explosive power. However, those of the male and female models indicated a significantly positive correlation; that is, a higher level of between-person flexibility signified a higher level of explosive power.

In the between-person association between muscular endurance and explosive power, the coefficients of the overall, male, and female models were b = 188.210, β = 0.628, *p* < 0.001; b = 58.788, β = 0.355, *p* < 0.001; and b = 54.344, β = 0.394, *p* < 0.001, respectively (Figure 3 and Figure 4). The results of all three models exhibited a significantly positive correlation in the relationship, thereby indicating that superior muscular endurance entails greater explosive power.

In the within-person association between BMI and explosive power, the coefficients of the overall, male, and female models were b = −0.839, βs = −0.050–−0.075, *p* < 0.001; b = −1.035, βs = −0.055–−0.083, *p* < 0.001; and b = −0.573, βs = −0.044–−0.056, *p* < 0.001, respectively (Figure 3 and Figure 4). These results revealed that in all three models, higher within-person BMI corresponded to inferior within-person explosive power.

In the within-person association between BMI and muscular endurance, the coefficients of the overall, male, and female models were b = −0.256, βs = −0.040–−0.070, *p* < 0.001; b = −0.202, βs = −0.029–−0.049, *p* < 0.001; and b = −0.310, βs = −0.052–−0.098, *p* < 0.001, respectively (Figure 3 and Figure 4), which signified that in all three models, higher within-person BMI was associated with lower within-person muscular endurance.

In the within-person association between BMI and flexibility, the coefficients of the overall, male, and female models were b = −0.303, βs = −0.058–−0.087, *p* < 0.001; b = −0.165, βs = −0.034–−0.047, *p* < 0.05; and b = −0.489, βs = −0.082–−0.142, *p* < 0.001, respectively (Figure 3 and Figure 4). This indicated that in all three models, higher within-person BMI corresponded to poorer within-person flexibility.

As for the within-person association between flexibility and muscular endurance, the coefficients of the overall, male, and female models were b = 2.119, βs = 0.061–0.135, *p* < 0.001; b = 2.275, βs = 0.062–0.119, *p* < 0.001; and b = 1.942, βs = 0.058–0.165, *p* < 0.001, respectively (Figure 3 and Figure 4). Such results demonstrated that in all three models, higher within-person flexibility suggested higher within-person muscular endurance.

As for the within-person relationship between flexibility and explosive power, the coefficients of the overall, male, and female models were b = 4.089, βs = 0.038–0.116, *p* < 0.001; b = 5.174, βs = 0.046–0.117, *p* < 0.001; and b = 2.205, βs = 0.021–0.089, *p* < 0.001, respectively (Figure 3 and Figure 4), which indicated that in all three models, higher within-person flexibility corresponded to greater within-person explosive power.

In the within-person association between muscular endurance and explosive power, the coefficients of the overall, male, and female models were b = 15.428, βs = 0.136–0.279, *p* < 0.001; b = 23.373, βs = 0.183–0.322, *p* < 0.001; and b = 5.948, βs = 0.059–0.177, *p* < 0.001, respectively (Figure 3 and Figure 4), showing that in all three models, higher within-person muscular endurance entailed greater within-person explosive power.

The BMI autoregressive coefficients of the overall, male, and female models were b = 0.054, βs = 0.059–0.112, *p* < 0.001; b = 0.073, βs = 0.039–0.142, *p* < 0.001; and b = 0.035, βs = 0.015–0.080, *p* < 0.05, respectively (Figure 3 and Figure 4), suggesting that BMI in all the models exhibited cross-time stability, meaning that BMI had carry-over effect. In other words, BMI in the past time will influence BMI in the present time and BMI in the present time will influence BMI in the future time.

The flexibility autoregressive coefficients of the overall, male, and female models were b = 0.018, βs = 0.013–0.032, *p* > 0.05; b = 0.028, βs = 0.021–0.046, *p* > 0.05; and b = 0.004, βs = 0.003–0.009, *p* > 0.05, respectively. This revealed that flexibility in the models exhibited no cross-time stability.

The muscular endurance autoregressive coefficients of the overall, male, and female models were b = 0.088, βs = 0.075–0.107, *p* < 0.001; b = 0.124, βs = 0.104–0.143, *p* < 0.001; and b = 0.040, βs = 0.030–0.055, *p* > 0.05, respectively, demonstrating that muscular endurance in the overall and male models, but not the female model, had cross-time stability.

The explosive power autoregressive coefficients of the overall, male and female models were b = 0.019, βs = 0.012–0.029, *p* > 0.05; b = 0.034, βs = 0.028–0.052, *p* > 0.05; and b = 0.019, βs = 0.010–0.040, *p* > 0.05, respectively. Explosive power in all models was not stable over time.

### Interaction Effect of Sex

**Figure 5:**
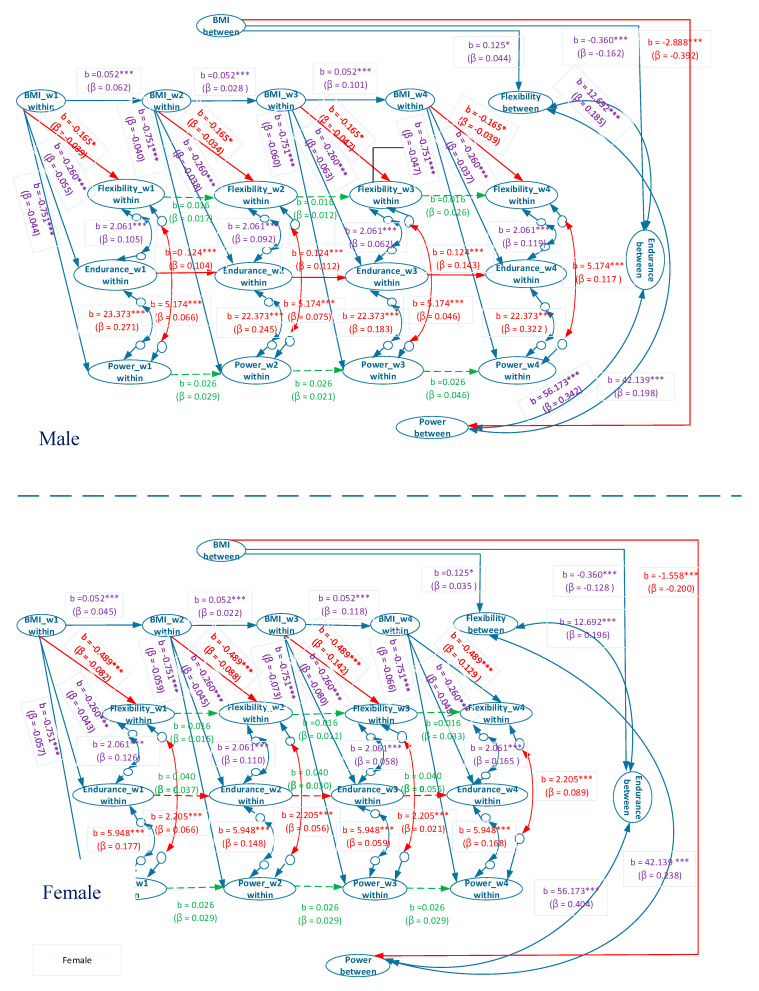
Simplified representation of the constrained multi-group random-intercept panel models for male and female participants.

Table 4 displays the fit indices of the multi-group sex RIPM examining the interaction effect of sex. The fit indices of the multi-group baseline model (M0) implied that the model was acceptable (χ^2^ (224) = 1833.93, *p* < 0.001, RMSEA = 0.068, CFI = 0.959, SRMR = 0.086). We established 16 equivalent models and compared the chi-square values of them and the baseline model. The Δ*x*^2^ values of five of the sixteen models (M3, M4, M9, M15, and M16) represented a significant difference, indicating that the five parameters signified an in-equivalence between sex (Table 4). As can be observed in Figure 5, the negative effect of between-person BMI on between-person explosive power for the male participants was significantly larger than that for the female participants (male: b = −2.888, female: b = −1.558) (Figure 5), whereas the negative effect of within-person BMI on within-person flexibility for the female participants was significantly stronger than that for the male participants (male: b = −0.165, female: b = −0.489) (Figure 5). The auto-regression of within-person muscular endurance for the male participants was significantly higher than that for the female participants (male: b = 0.124, female: b = 0.040) (Figure 5); the within-person muscular endurance of the male participants presented a carry-over effect that was absent for the female participants. The within-person flexibility and within-person explosive power of the male participants were significantly and positively correlated, as well as stronger than that for the female participants (male: b = 5.174, βs = 0.046–0.117; female: b = 2.205, βs = 0.021–0.089) (Figure 5) because the within-person flexibility and within-person explosive power of the female participants were not correlated. The within-person muscular endurance and within-person explosive power of both sexes exhibited significant correlations (male: b = 23.373, βs = 0.183–0.322; female: b = 5.948, βs = 0.059–0.177) (Figure 5), although the correlation for the male participants was significantly stronger than that of the female participants.

The Δ*x*^2^ values of the remaining 11 equivalent models did not reach levels of significance, implying that the male and female participants can be viewed as equivalents for them (Table 3). Male and female participants had equivalent between-person relationships between BMI and flexibility, which were significantly and positively correlated (b = 0.125) (Figure 5); they had equivalent between- and within-person relationships between BMI and muscular endurance, which were significantly and negatively correlated (b = −0.360 and b = −0.260, respectively) (Figure 5); they had equivalent within-person relationships between BMI and explosive power, which were significantly and negatively correlated (b = −0.751). Furthermore, the auto-regression of within-person BMI of male and female participants were equivalent and demonstrated a significant, positive effect (b = 0.052) (Figure 5); the auto-regressions of within-person flexibility and within-person explosive power of male and female participants yielded equivalent results but revealed no significant influences (b = 0.016, b = 0.026) (Figure 5). The between-person correlations between flexibility and explosive power, between flexibility and muscular endurance, between muscular endurance and explosive power, and the within-person correlation between flexibility and muscular endurance were all equivalent and significantly and positively correlated in both the male and female participants (b = 42.139, b = 12.692, b = 56.173, and b = 2.061, respectively) (Figure 5).

## 4. Discussion

Studies comparing the physical fitness of young adults with normal and overweight/obese weight statuses using cross-sectional data have consistently identified a negative relationship between them [13,18,21]. The present study applied longitudinal data (in four waves) to examine relationships between BMI, flexibility, muscular endurance, and explosive power, and employed a RIPM to divide the variations of different waves into between- and within-person variations. Preceding studies investigating the interaction effect of sex on between- and within-person relationships between BMI, flexibility, muscular endurance, and explosive power have not conducted tests for statistical significance. The present study therefore incorporated a multi-group sex invariance test to more accurately examine the relationships.

Because variations were divided into between- and within-person variations, notable results among the overall, male, and female models and the invariance test were observed. First, Simpson’s paradox was observed in the between-person relationships between BMI and flexibility, BMI and muscular endurance, and BMI and explosive power in all three models, which sustained the legitimacy of preceding studies in examining male and female participants separately despite the fact that they did not propose reasonable arguments for such an approach. This also implied that studies that have not separately investigated male and female samples may have reached false conclusions.

Furthermore, this study discovered a negative between-person relationship between BMI and explosive power, independent of sex, which corresponded with the results of Huang and Malina [32] and Lopes et al. [20,33]. These studies adopted standing long jump to measure explosive power, and the results revealed that, independent of sex, BMI exceeding normal weight status has a negative correlation with performance, which accorded with the result of the present study. The theory of Voelkle et al. [34] that the mechanism mostly occurs in a within-person relationship was supported; this study affirmed that for university students with normal/overweight/obese weight status, BMI and explosive power were negatively correlated. However, the sex invariance test implemented disclosed a different result. The negative between-person associations between BMI and explosive power were not equivalent between male and female participants. The association was significantly stronger among male participants, yet the within-person associations between BMI and explosive power were equivalent for both sexes. For these contradicting results, we accept the argument of Voelkle et al. [34] that within-person variance is more credible because it is a type of trait-like stability that excludes between-person variation. Therefore, we tend to accept that the association between BMI and explosive power are equal for both sexes.

This study also uncovered that between-person BMI and between-person muscular endurance, in both male and female participants, were negatively correlated; as between-person BMI increased, performance in between-person muscular endurance decreased, a finding similar to those of Bovet et al. [11] and Kwieciński et al. [21]. Although these studies have noted a quadratic relationship between BMI and sit-ups, the curve indicated that the peak value falls close to the starting point of average weight status; sit-up performance then declines as BMI increases. The research of Mak et al. [17] also implied that first, overweight or obese boys and girls perform worse in sit-ups compared with those with a normal weight status and second, that the within-person BMI and within-person muscular endurance of both boys and girls were negatively correlated. We similarly concluded that the BMI and muscular endurance of university students with normal/overweight/obese weight status exhibited a negative correlation.

Regarding the strength of the relationship between muscular endurance and BMI for boys and girls separately, Liao et al. [12] demonstrated that correlations between BMI and sit-ups for boys and girls were −0.03 (*p* < 0.05) and −0.02 (*p* > 0.05), respectively. Kwieciński et al. [21] claimed in their report that in contrast to other fitness items, the distribution of performances of girls on the sit-up test has a more parabolic shape than that of boys, which implied that the strength of the relationship differs between boys and girls. Nevertheless, our invariance test verified that the negative between- and within-person correlations between BMI and muscular endurance were both invariant with respect to sex; hence, their strengths should be viewed as equal.

The results of the present study demonstrated that the between-person associations between BMI and flexibility for males was positive but those of female were not. These results are consistent with the study of Mak et al. [17]. They concluded that the BMI and flexibility of boys are positively correlated but those of girls are not. However, the results of the present study revealed that the associations between BMI and flexibility at the within-person level were negative among both male and female participants. Furthermore, the invariance test confirmed that the positive correlation of between-person BMI and between-person flexibility was invariant between sexes, yet the negative correlation of within-person BMI and within-person flexibility was not equivalent between sexes; specifically, the correlation was stronger among female participants than male ones. According to the argument of Voelkle et al. [34], a negative within-person correlation between BMI and flexibility is more credible because this variation is a type of trait-like stability that excludes between-person variation. The non-invariant result of the negative correlation for male and female participants (female participants with a stronger correlation than male participants) is more consistent with theoretical expectations regarding relationships between sex, BMI, and physical fitness.

This study indicated that between- and within-person associations between explosive power and muscular endurance of both sexes were positive, which accorded with theoretical expectations because these two physical fitness items are related to muscle strength. The results also implied that the strength of the relationships did not vary by sex. The results were different from those found in Yanci, Los Arcos, Castillo, and Cámara’s study [35]. They found that in groups under 12 years of age and under 14 years of age, significant gender differences were observed in the modified agility test (MAT). Likewise, boys under 16 years of age obtained better results than girls under 16 years of age in the horizontal jump tests.

Between- and within-person correlations among explosive power, muscular endurance, and flexibility were positive in this study, independent of sex. This corresponded to the results of preceding studies. Mak et al. [17] claimed that the muscular endurance and flexibility of both boys and girls are positively correlated. Aphamis et al. [36] asserted that a positive correlation exists between handgrip and vertical jump, and also 30 m sprint time and VO_2_ max (maximal oxygen uptake) based on participants’ performance in strength tests. He et al. [37] concluded that the performances of both sexes in hand grip strength, vertical jump, and sit-and-reach are significantly correlated.

In the auto-regression of BMI, flexibility, muscular endurance, and explosive power, only BMI and muscular endurance were significant and equivalent between the sexes. This result implied that carry-over effects of BMI and muscular endurance existed (a carry-over effect existed between BMI and muscular endurance) and accorded with that of Eichler et al. [38]. Eichler et al. applied a long-term cross lagged panel model to examine the stability of BMI, and the results suggested that BMI between z-scores exhibited high stability. Regarding physical fitness items, only the research of Lefevre et al. [39] was suitable for a comparison with the present study. A simplex model was employed in their research to explore the stability of physical fitness items. Sit and reach, vertical jump, and bent arm hang were respectively applied for examinations of flexibility, explosive power, and upper limb muscle endurance, and the results revealed that these items have considerable stability.

### Limitations and Suggestions for Future Studies

Despite the numerous advantages of this study, limitations were identified. First, university students with normal, overweight, or obese weight status were selected, and the results are not generalizable to those who are underweight. In addition, recruitment began at the start of the first semester and ended at the end of the second, and it was implemented once every three months. The results present temporal changes in BMI over four waves (summer, fall, winter, and spring); however, this does not imply an optimal time interval for recruitment. The relationship between BMI and physical fitness is a complex and dynamic process; accordingly, Frijns et al. [40] proposed that long-term effects do not necessarily correspond to short-term effects, namely the galloping horse fallacy. The duration of a study period may lead to different results; hence, caution is required in the generalization of the results proposed by this study.

## 5. Conclusions

This study aimed to employ four-wave longitudinal data to examine relationships between BMI, flexibility, muscular endurance, and explosive power, and utilized a random intercept panel model (RIPM) to decompose the variations of different waves into between- and within-person variations. Furthermore, a multi-group model test was conducted to explore whether an interaction effect existed between sex and these relationships. Our results showed that the associations between BMI and explosive power, and between BMI and muscular endurance was negative in both between- and within-person levels, independent of sex. The invariance test verified that the negative between- and within-person correlations between BMI and muscular endurance were both invariant regarding sex. The negative between-person correlation between BMI and explosive power for males and females was not equivalent, and the correlation was significantly stronger among male participants. Nevertheless, the within-person correlation between BMI and explosive power was equivalent for both sexes. The between-person association between BMI and flexibility of male and female participants both were positive, but the within-person associations of these two variables were negative for both males and females. The invariance test verified that the positive correlation of between-person BMI and between-person flexibility was invariant between sexes, yet the negative correlation of within-person BMI and within-person flexibility was not equivalent between sexes. Between- and within-person correlations among explosive power, muscular endurance, and flexibility were positive in this study. In addition, our results showed that in the auto-regression of BMI, flexibility, muscular endurance, and explosive power, only BMI and muscular endurance were significant and equivalent between the sexes. The findings from this research contribute to practitioners and researchers to deeply investigate the potential mechanism between BMI and physical fitness.

## Figures and Tables

**Table 1:** Demographics of the participants.

Variables	Category	N	%
Sex	Male	1661	53.1
	Female	1465	46.9
Colleges	Management	421	13.3
	Humanities and Arts	466	14.9
	Normal	421	13.5
	Agricultural	679	21.7
	Science and Engineering	682	21.8
	Life sciences	461	14.7
Grade	Freshman	1643	52.6
	Junior	1483	47.4
Age (years)	17	8	0.3
	18	1330	42.5
	19	1398	44.7
	20	303	9.7
	21	52	1.7
	22	18	0.6
	23	17	0.5

**Table 2:** Means, standard deviations (SD), and sample size (N) of main variables.

	Male (*n* = 1661)				Female (*n* = 1465)				t Value (*p*)
	Mean	SD	Available N	(% of All)	Mean	SD	Available N	(% of All)	
1. BMI_W1	22.93	3.56	1447	87.12%	21.68	2.69	1283	87.58%	10.29 (<0.001)
2. BMI_W2	22.74	3.36	1197	72.07%	21.74	2.63	1181	80.61%	8.04 (<0.001)
3. BMI_W3	22.87	3.32	1500	90.31%	21.94	2.77	1308	89.28%	8.01 (<0.001)
4. BMI_W4	22.84	3.44	1387	83.50%	21.74	2.73	1098	74.95%	8.64 (<0.001)
5. Flexibility_W1	28.55	10.52	1544	92.96%	34.44	10.20	1453	99.18%	−15.54 (<0.001)
6. Flexibility_W2	26.26	10.16	1250	75.26%	32.39	9.63	1260	86.01%	−15.51 (<0.001)
7. Flexibility_W3	26.79	10.05	1550	93.32%	32.05	9.67	1355	92.49%	−14.32 (<0.001)
8. Flexibility_W4	27.89	10.28	1475	88.80%	33.56	9.84	1208	82.46%	−14.49 (<0.001)
9. Endurance_W1	41.53	8.84	1544	92.96%	31.59	8.19	1453	99.18%	31.89 (<0.001)
10. Endurance_W2	41.41	9.28	1249	75.20%	31.60	8.56	1258	85.87%	27.51 (<0.001)
11. Endurance_W3	40.09	9.73	1549	93.26%	31.80	8.45	1352	92.29%	24.34 (<0.001)
12. Endurance_W4	41.45	9.49	1475	88.80%	32.10	8.31	1207	82.39%	26.82 (<0.001)
13. Power _W1	201.87	29.72	1544	92.96%	149.66	21.65	1453	99.18%	54.69 (<0.001)
14. Power_W2	203.88	28.21	1247	75.08%	149.98	22.09	1256	85.73%	53.24 (<0.001)
15. Power_W3	199.24	31.64	1548	93.20%	150.59	25.53	1346	91.88%	45.07 (<0.001)
16. Power_W4	202.41	27.88	1474	88.74%	148.13	22.12	1207	82.39%	54.95 (<0.001)

Note: w1, wave one; w2, wave two; w3, wave three; w4, wave four.

**Table 3:** Coefficients of BMI, flexibility, muscular endurance, and explosive power with normal/overweight/obese weight status and inter-class correlation coefficients (ICCs).

	1.	2.	3.	4.	5.	6.	7.	8	9.	10.	11.	12.	13.	14	15.	16.
1. BMI_W1	1	0.94 **	0.76 **	0.94 **	0.03	0.01	0.01	0.02	−0.09 **	−0.13 **	−0.05	−0.10 **	−0.18 **	−0.20 **	−0.14 **	−0.18 **
2. BMI_W2	0.93 **	1	0.75 **	0.92 **	0.05	0.01	0.03	0.06	−0.06 *	−0.10 **	−0.03	−0.07 *	−0.19 **	−0.19 **	−0.15 **	−0.19 **
3. BMI_W3	0.84 **	0.84 **	1	0.79 **	0.02	0.01	−0.03	0.02	−0.02	−0.05	−0.06 *	−0.02	−0.13 **	−0.14 **	−0.12 **	−0.14 **
4. BMI_W4	0.93 **	0.94 **	0.89 **	1	0.02	0.02	−0.01	0.01	−0.07 *	−0.09 **	−0.03	−0.09 **	−0.16 **	−0.18 **	−0.11 **	−0.18 **
5. Flexibility_W1	0.06 *	0.03	0.07 **	0.06 *	1	0.86 **	0.73 **	0.90 **	0.17 **	0.12 **	0.11 **	0.17 **	0.19 **	0.19 **	0.13 **	0.17 **
6. Flexibility_W2	0.03	−0.01	0.05	0.01	0.84 **	1	0.74 **	0.86 **	0.14 **	0.15 **	0.10 **	0.17 **	0.19 **	0.21 **	0.12 **	0.17 **
7. Flexibility_W3	0.04	−0.02	0.01	0.02	0.74 **	0.78 **	1	0.79 **	0.11 **	0.09 **	0.13 **	0.17 **	0.15 **	0.14 **	0.10 **	0.15 **
8. Flexibility_W4	0.07 *	0.04	0.06 *	0.04	0.87 **	0.85 **	0.80 **	1	0.16 **	0.13 **	0.14 **	0.19 **	0.19 **	0.19 **	0.14 **	0.20 **
9. Endurance_W1	−0.17 **	−0.14 **	−0.14 **	−0.15 **	0.16 **	0.17 **	0.12 **	0.15 **	1	0.79 **	0.62 **	0.78 **	0.35 **	0.35 **	0.26 **	0.34 **
10. Endurance_W2	−0.15 **	−0.14 **	−0.12 **	−0.15 **	0.13 **	0.19 **	0.14 **	0.15 **	0.69 **	1	0.63 **	0.75 **	0.34 **	0.37 **	0.24 **	0.33 **
11. Endurance_W3	−0.15 **	−0.13 **	−0.14 **	−0.14 **	0.11 **	0.16 **	0.14 **	0.14 **	0.57 **	0.59 **	1	0.66 **	0.26 **	0.25 **	0.39 **	0.27 **
12. Endurance_W4	−0.15 **	−0.14 **	−0.15 **	−0.16 **	0.16 **	0.19 **	0.14 **	0.18 **	0.70 **	0.65 **	0.67 **	1	0.34 **	0.33 **	0.24 **	0.34 **
13. Power _W1	−0.36 **	−0.32 **	−0.30 **	−0.32 **	0.17 **	0.13 **	0.14 **	0.14 **	0.33 **	0.21 **	0.22 **	0.24 **	1	0.84 **	0.63 **	0.85 **
14. Power_W2	−0.35 **	−0.32 **	−0.29 **	−0.32 **	0.12 **	0.17 **	0.15 **	0.14 **	0.24 **	0.30 **	0.25 **	0.26 **	0.66 **	1	0.63 **	0.84 **
15. Power_W3	−0.27 **	−0.24 **	−0.27 **	−0.26 **	0.15 **	0.15 **	0.13 **	0.16 **	0.24 **	0.28 **	0.45 **	0.29 **	0.57 **	0.60 **	1	0.67 **
16. Power_W4	−0.35 **	−0.34 **	−0.31 **	−0.33 **	0.15 **	0.16 **	0.15 **	0.15 **	0.26 **	0.27 **	0.27 **	0.32 **	0.72 **	0.74 **	0.71 **	1
	Male								Female							
	BMI	Flexibility		Endurance		Power			BMI	Flexibility		Endurance		Power		
ICC(1)	0.90	0.81		0.65		0.67			0.84	0.80		0.70		0.72		

Note: The upper and lower triangular areas display the female and male samples, respectively; * *p* < 0.05; ** *p* < 0.01.

**Table 4:** Fit indices for a multi-group random-intercept panel model (RIPM) of sex for overweight and normal weight samples.

Model	χ^2^	df	Comp.	Δχ^2^	Δdf	*p* value	RMSEA	CFI	SRMR
M0	1833.93	224					0.068	0.959	0.086
M1 (bmib -> fleb)	1834.09	225	M1-M0	0.16	1	0.689	0.068	0.959	0.086
M2 (bmib -> endb)	1836.72	225	M2-M0	2.79	1	0.095	0.068	0.959	0.086
M3 (bmib -> powb)	1856.48	225	M3-M0	22.55 *	1	<0.001	0.068	0.959	0.091
M4 (bmiw -> flexw)	1843.87	225	M4-M0	9.94 *	1 ^a^	0.002	0.068	0.958	0.086
M5 (bmiw -> endw)	1834.80	225	M5-M0	0.87	1 ^b^	0.351	0.068	0.959	0.086
M6 (bmiw -> poww)	1835.91	225	M6-M0	1.98	1 ^c^	0.159	0.068	0.959	0.086
M7 (bmiw auto)	1836.14	225	M7-M0	2.21	1 ^d^	0.137	0.068	0.959	0.086
M8 (flexw auto)	1834.62	225	M8-M0	0.69	1 ^e^	0.406	0.068	0.959	0.086
M9 (endw auto)	1840.03	225	M9-M0	6.10 *	1 ^f^	0.014	0.068	0.959	0.086
M10 (poww auto)	1834.26	225	M10-M0	0.33	1 ^g^	0.566	0.068	0.959	0.086
M11 (fleb_endb_r)	1835.31	225	M11-M0	1.38	1	0.240	0.068	0.959	0.086
M12 (fleb_powb_r)	1834.43	225	M12-M0	0.50	1	0.480	0.068	0.959	0.086
M13 (endb_powb_r)	1834.37	225	M13-M0	0.44	1	0.507	0.068	0.959	0.086
M14 (flew_endw_r)	1834.92	225	M14-M0	0.99	1 ^h^	0.320	0.067	0.959	0.086
M15 (flew_poww_r)	1840.14	225	M15-M0	6.21 *	1 ^i^	0.012	0.068	0.959	0.086
M16 (endw_poww_r)	1937.95	225	M16-M0	104.02 *	1 ^j^	<0.001	0.070	0.956	0.085

Note: a, b, c, d, e, f, g, h, i, j: Due to equality constraints in the baseline RIPM (M0), the change of df is 1. bmib, between-level BMI; fleb, between-level flexibility; endb, between-level muscular endurance; powb, between-level explosive power; bmiw, within-level BMI; flew, within-level flexibility; endw, within-level muscular endurance; poww, within-level explosive power; auto, autoregression; r, correlation; **χ^2^**, chi-squared; RMSEA, Root mean square error of approximation; CFI, Comparative-Fit Index; SRMR, Standardized root mean square residual; * *p* < 0.05.
